# Carnivore conservation needs evidence-based livestock protection

**DOI:** 10.1371/journal.pbio.2005577

**Published:** 2018-09-18

**Authors:** Lily M. van Eeden, Ann Eklund, Jennifer R. B. Miller, José Vicente López-Bao, Guillaume Chapron, Mikael R. Cejtin, Mathew S. Crowther, Christopher R. Dickman, Jens Frank, Miha Krofel, David W. Macdonald, Jeannine McManus, Tara K. Meyer, Arthur D. Middleton, Thomas M. Newsome, William J. Ripple, Euan G. Ritchie, Oswald J. Schmitz, Kelly J. Stoner, Mahdieh Tourani, Adrian Treves

**Affiliations:** 1 Desert Ecology Research Group, School of Life and Environmental Sciences, University of Sydney, Camperdown, Australia; 2 Grimsö Wildlife Research Station, Department of Ecology, Swedish University of Agricultural Sciences, Riddarhyttan, Sweden; 3 Department of Environmental Science, Policy, and Management, University of California–Berkeley, Berkeley, California, United States of America; 4 Center for Conservation Innovation, Defenders of Wildlife, Washington, DC, United States of America; 5 Research Unit of Biodiversity, Oviedo University, Gonzalo Gutiérrez Quirós, Mieres, Spain; 6 Department of Natural Sciences, Paul Smith’s College, Paul Smiths, New York, United States of America; 7 Lake Placid Land Conservancy, Lake Placid, New York, United States of America; 8 Biotechnical Faculty, Department of Forestry, University of Ljubljana, Ljubljana, Slovenia; 9 Wildlife Conservation Research Unit, Department of Zoology, University of Oxford, The Recanati-Kaplan Centre, Tubney House, Tubney, Abingdon, United Kingdom; 10 Research Department, Landmark Foundation, Riversdale, South Africa; 11 School of Animal, Plants and Environmental Sciences, University of Witwatersrand, Braamfontein, Johannesburg, South Africa; 12 Yale School of Forestry and Environmental Studies, New Haven, Connecticut, United States of America; 13 School of Environmental and Forest Sciences, University of Washington, Seattle, Washington, United States of America; 14 Global Trophic Cascades Program, Department of Forest Ecosystems and Society, Oregon State University, Corvallis, Oregon, United States of America; 15 Centre for Integrative Ecology, School of Life and Environmental Sciences, Deakin University, Burwood, Victoria, Australia; 16 Wildlife Conservation Society Rocky Mountain Regional Program, Bozeman, Montana, United States of America; 17 Faculty of Environmental Sciences and Natural Resource Management, Norwegian University of Life Sciences, Ås, Norway; 18 Nelson Institute for Environmental Studies, University of Wisconsin, Madison, Wisconsin, United States of America

## Abstract

Carnivore predation on livestock often leads people to retaliate. Persecution by humans has contributed strongly to global endangerment of carnivores. Preventing livestock losses would help to achieve three goals common to many human societies: preserve nature, protect animal welfare, and safeguard human livelihoods. Between 2016 and 2018, four independent reviews evaluated >40 years of research on lethal and nonlethal interventions for reducing predation on livestock. From 114 studies, we find a striking conclusion: scarce quantitative comparisons of interventions and scarce comparisons against experimental controls preclude strong inference about the effectiveness of methods. For wise investment of public resources in protecting livestock and carnivores, evidence of effectiveness should be a prerequisite to policy making or large-scale funding of any method or, at a minimum, should be measured during implementation. An appropriate evidence base is needed, and we recommend a coalition of scientists and managers be formed to establish and encourage use of consistent standards in future experimental evaluations.

Carnivores, such as lions and wolves, are killed in many regions over real or perceived threats to human interests. Combined with habitat loss and fragmentation, human-induced mortality has contributed to widespread carnivore population declines, along with declines of their important ecosystem functions [1]. Balancing the goals of nature preservation, livelihood protection, and welfare of carnivores and domestic animals depends on policies that foster coexistence between humans and carnivores in multiuse landscapes [2, 3]. Central to this aim is a need for rigorous scientific evidence that interventions are effective in preventing predation on livestock. Such policies should be based on strong inference [4, 5], otherwise, we risk wasting resources on ineffective interventions that might harm all involved.

Between 2016 and 2018, we independently published four reviews examining evidence for the effectiveness of interventions to reduce livestock predation by carnivores [6–9]. Here, we focus on the results for livestock losses or carnivore incursions into livestock enclosures (hereafter, “functional effectiveness” [8]). Since each review offered a unique perspective, we reconcile differences to synthesize three messages common to the reviews. First, despite the immense resources spent globally to protect livestock from carnivores, few peer-reviewed studies have produced strong inference about the functional effectiveness of interventions. Second, there was scant consistency of standards of evidence in our four reviews, hindering scientific consensus, and hence clear recommendations to policy-makers, about the relative functional effectiveness of different interventions. Finally, we identified several interventions that were found consistently effective, which deserve promotion in policy, even if only in the general conditions under which they have already been tested, as well as prioritization for further research under conditions in which evidence is lacking.

We suspect that the striking paucity of rigorous evaluation is due to the tendency for decisions about predator control to depend on factors other than evidence-based evaluation of whether a given intervention effectively protects livestock. These other factors—including ethics (should one implement the intervention?), feasibility (can one implement the intervention?), and perception (does one believe the intervention will work?)—might be important subsequent considerations in the implementation and decision-making processes. However, objective scientific evidence of an intervention’s functional effectiveness must remain a foundational prerequisite on which subjective inquiries later build. The lack of scientific synthesis and consensus about functional effectiveness has allowed more subjective factors to dominate decision-making about predator control and likely wasted time and money on interventions that do not optimally protect livestock. Furthermore, shifting ethics and public values in some communities are enabling the return of carnivores to landscapes worldwide or leading to the increased use of nonlethal predator control interventions. We support these initiatives from the perspective of conserving carnivores but insist that scientific evidence for functional effectiveness be considered first to ensure that interventions intended to protect livestock accomplish that goal. This will prevent the inefficient—or worse yet, counterproductive—use of limited resources to protect animals long term.

Additionally, although our reviews collectively reveal a need for more evidence, scientists alone cannot fill this gap. Livestock owners, natural resource managers, and decision-makers each have an important role to play in research partnerships to collaboratively guide the testing of predator control interventions. Here, we appeal to these groups by summarizing the advantages of evidence-based effective interventions, the best practices of scientific inference, and the role of policy in promoting effective predator control strategies. We start by synthesizing the results of our four independent reviews to provide scientific consensus on the evaluations of predator control interventions. We urge managers and policy decision-makers to use this discussion as a basis for creating policy that promotes evidence-based, effective strategies for protecting domestic animals from carnivore predation.

## Synthesis of the science on functional effectiveness

Our four reviews [6–9] jointly screened >27,000 candidate studies. The four sets of inclusion criteria differed in geographic coverage, carnivore species, and standards of evidence and research design (see S1 Table), which limited overlap in the studies that passed screening (only 19% of studies were included in two or more of the four reviews; no study was included in all four, S1 Fig). The differing inclusion criteria also meant that it was not possible to conduct a quantitative comparison (meta-analysis) combining the data from our four reviews, but we suggest that such an analysis should be conducted in the future as evidence increases. Nonetheless, our reviews came to remarkably similar conclusions, irrespective of methods, suggesting that our conclusions are robust.

Among the 114 studies that passed screening in one or more reviews (S2 Table), representing >40 years of research, we found few that yielded strong inference about functional effectiveness. Surprisingly, many widely used methods have not been evaluated using controlled experiments. Also, few interventions have been compared side by side or tested singly under diverse conditions. These deficiencies in the literature are further compounded by disagreement among scientists, managers, and peer-reviewed journals about standards of evidence, such as which study designs produce strong inference [8]. We acknowledge the challenges of regional experiments amid dynamic, complex ecologies, publics, and jurisdictions. However, a handful of random-assignment experimental studies without bias (“gold standard”) have proven that the obstacles are surmountable [8, 10, 11, 12].

**Fig 1:**
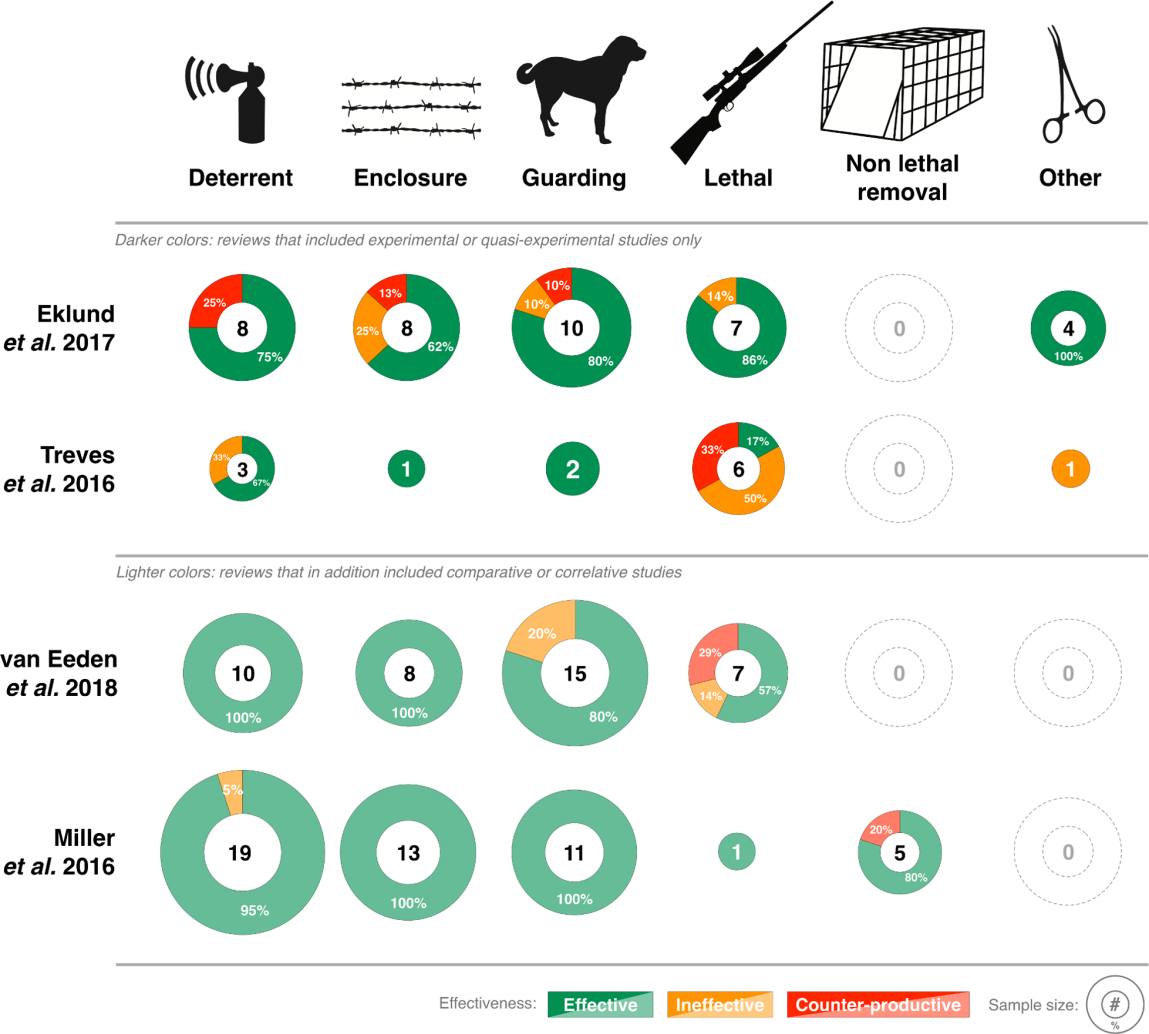
Percent of studies that measured interventions as “Effective,” “Ineffective,” or “Counter-productive” in reducing livestock loss to large carnivores, as measured by four independent reviews in 2016–2018. The sample sizes inside disks represent the number of studies or tests, as some studies reported more than one test of the same or different interventions. Darker colors represent reviews that included experimental or quasiexperimental controls; lighter colors represent reviews that also included comparative or correlative studies (see S1 Table for details). “Deterrents” include nonlethal interventions such as audio or visual deterrents, fladry, and livestock protection collars. “Enclosure/barrier” includes electrified and nonelectrified fencing and corralling. “Guarding” includes human shepherding and livestock guardian animals. “Lethal removal” includes hunting, poison baiting, and other lethal methods. “Non-lethal removal” refers to translocation of carnivores. “Other” includes carnivore sterilization and diversionary feeding. Eklund and colleagues measured effectiveness using RR and classified Effective as RR < 0.90, Ineffective = 0.90–1.10, and Counterproductive RR > 1.10. Treves and colleagues measured effectiveness as significant change in livestock loss. Note that Treves and colleagues initially contained 12 studies with 14 separate tests using gold or silver standards, but one test was subsequently removed after review of the methods found it impossible to draw strong inference [17]. van Eeden and colleagues measured effectiveness as Hedges’ *d* and classified Effective as *d* < −0.05, Ineffective −0.05 > *d* < 0.05, and Counterproductive *d* > 0.05. Miller and colleagues measured effectiveness as percentage change in livestock loss (or carnivore behavior change) and classified Effective as *d* > 0% change, Ineffective = 0%, and Counterproductive < 0%. RR, relative risk.

**Fig 2:**
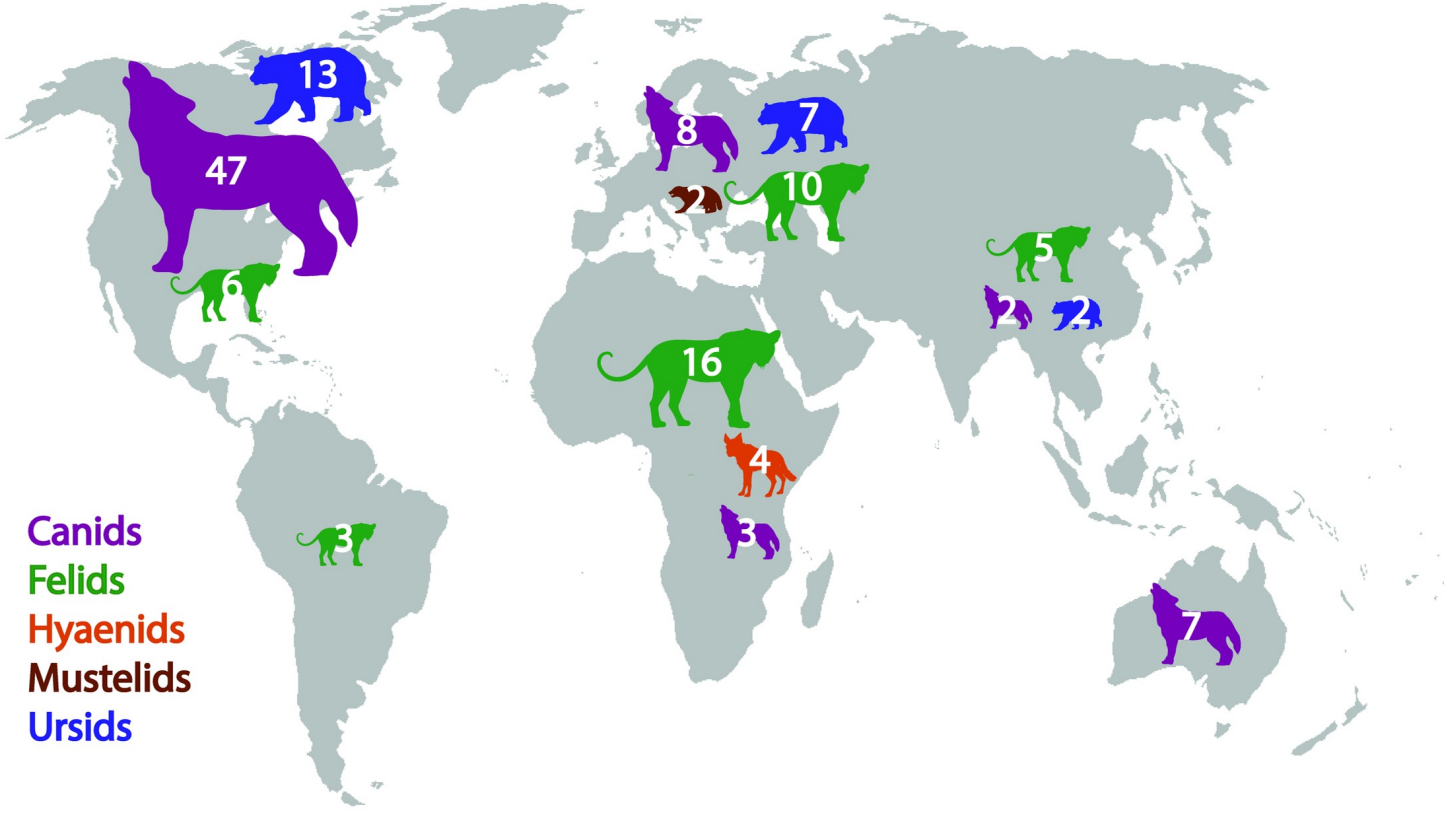
Number of studies included in four independent reviews published in 2016–2018, presented by carnivore family and continent. Canids include gray wolves and subspecies (*Canis lupus*), coyotes (*C*. *latrans*), dingoes (*C*. *dingo*), black-backed jackals (*C*. *mesomelas*), African wild dogs (*Lycaon pictus*), red foxes (*Vulpes vulpes*), and domestic dogs (*C*. *familiaris*). Felids include Eurasian lynx (*Lynx lynx*), cougars (*Puma concolor*), lions (*Panthera leo*), jaguars (*P*. *onca*), leopards (*P*. *pardus*), snow leopards (*P*. *uncia*), caracals (*Caracal caracal*), and cheetahs (*Acinonyx jubatus*). Hyaenids include spotted hyenas (*Crocuta crocuta*). Mustelids feature wolverines (*Gulo gulo*). Ursids include American black bears (*Ursus americanus*), Asiatic black bears (*U*. *thibetanus*), brown or grizzly bears (*U*. *arctos*), and polar bears (*U*. *maritimus*). Smaller carnivores (e.g., red foxes, hyenas, and caracals) are included in studies that investigated multiple carnivore species of varying sizes.

We summarize our four sets of results by category of intervention in Fig 1. Our reviews agree that several methods have been tested numerous times with high standards of evidence and have been found effective: livestock guardian animals, enclosures for livestock, and a visual deterrent called fladry. Importantly, we should recognize that the effectiveness of different methods will vary under different contexts, and there is currently a bias among research toward certain geographic regions and predator types (Fig 2). Further, we agree that standards of evidence have been higher for nonlethal methods, and there remains a need to ensure data on all interventions are collected appropriately and consistently. As such, building on existing criticism of the lack of appropriate data collection in environmental management [13–16], our reviews collectively highlight the need to improve standards of evidence used in evaluating interventions. We need to develop a comprehensive evidence base that allows us to compare the effectiveness of interventions for reducing carnivore predation on livestock and inform consistent policy in any jurisdiction.

## Importance of rigorous experimental design and evaluation

Societal values and, accordingly, policies for human–carnivore coexistence have changed over the millennia. The almost exclusive use of lethal interventions has given way to nonlethal interventions as important supplements to or replacements for prior lethal methods. Immense logistical and financial resources are invested in protecting livestock and carnivores, so the scarcity of rigorous scientific evidence for effectiveness should be a concern. We encourage governments to adopt proven methods from similar systems of carnivores and human interests, with systems in place to review and adapt management actions as new evidence becomes available. When governments contemplate large-scale implementation or funding for interventions, scientific evidence of functional effectiveness deserves priority to avoid wasting resources on ineffective methods, no matter if the latter are ethical or easy to implement. When no proven method is available, scientific evaluation of functional effectiveness should coincide with implementation.

Strong inference in any scientific field demands control over potentially confounding variables and testable claims about functional effectiveness of interventions [8]. In our context, all methods present opposable hypotheses, i.e., method X works or does not work. Several experimental design components are essential to strong inference about that hypothesis, and we focus here on the three of topmost priority for yielding strong inference about livestock protection interventions: controls, randomization, and replication.

The strongest inference results from experiments that achieve the “gold standard” through “random assignment to control and treatment groups without bias (systematic error) in sampling, treatment, measurement, or reporting” [8]. This requires that an intervention be used to protect a livestock herd (treatment) and that its effectiveness is compared against a livestock herd that is not exposed to the intervention (placebo control). Both treatment and control should be replicated using multiple independent herds of livestock that are distributed so that the effects of treatment on one herd do not confound the effects on another herd, which would eliminate independence. Random assignment of treatments avoids sampling or selection bias that is common in our field [8], as in others [18]. Implementing random assignment for actual livestock herds can be challenging, but several studies have succeeded, such as those conducted by Davidson-Nelson and Gehring [10] and Gehring and colleagues [11]. In the Chilean altiplano, 11 owners of alpacas (*Vicugna pacos*) and llamas (*Lama glama*) joined a randomized reverse treatment (crossover) experiment to evaluate light devices in deterring carnivores [12]. Moreover, if large numbers of replicates are infeasible or replicates are unavoidably heterogeneous, then crossover, reverse treatment designs should help to increase the strength of inference about interventions [8, 12, S2 Table].

“Silver standard” designs provide weaker inference because of nonrandom assignment to treatment and then repeated measures of the replicate at two or more time points (before-and-after comparison of impact or quasiexperimental designs, also called case control). Both time passing and the treatment might explain changes in replicates, in addition to the extraneous “nuisance” variables present in agro-ecosystems at the outset [8].

The weakest standard of evidence is the correlative study, which compares livestock predation among herds that varied haphazardly in past protection or varied systematically if people intervened only where livestock had died. In correlational studies, confounding variables inevitably create selection or sampling bias. Although correlative studies may be useful as an initial exploratory step and help direct further research, confidence in their findings should be low, especially if there is large variation in the results. Correlative studies cannot substitute for the silver or gold standards described above.

Implementation of interventions must be consistent to avoid treatment bias. For example, the functional effectiveness of livestock-guarding dogs might vary with breed, individual, training, and maintenance of the dog. Likewise, tests of lethal methods have never controlled the simultaneous use of several methods of intervention (e.g., pooling shooting and trapping as one treatment), which is inadvisable for strong inference. Consistent maintenance of interventions throughout a study should also minimize treatment bias [18].

Well-designed experiments should incorporate evaluation along multiple dimensions. Was the intervention implemented as planned? Did attacks on livestock diminish? Measurement bias arises from systematic error in documenting implementation or losses in treatment or response variables. As in biomedical research, which sometimes uses patient self-reports as a subjective measure of effectiveness alongside objective measures of health outcomes, there are valid reasons to measure owners’ perceptions of effectiveness of interventions. In human–wildlife interactions, people’s attitudes can influence the adoption or rejection of interventions independently of scientific evidence [14,19]. Several of the reviews included metrics of perceived effectiveness among livestock owners, yet perception alone is not a reliable measure of functional effectiveness because of widespread placebo effects, whereby patients feel better simply because they have participated. Studies should therefore either “blind” their participants or use an independent, verifiable measure of effectiveness (i.e., livestock loss).

We recognize that gold or silver standards may be difficult to achieve. Systematic errors can be difficult to eliminate entirely, so we urge careful consideration of methods during the design process, including peer review prior to initiation. Ethical considerations about exposing animals to lethal risks may limit experimental designs. This inherent difficulty for controlled experiments may explain why some published experiments were completed in artificial settings (e.g., using captive carnivores or measuring bait consumption rather than livestock loss). Although most of our reviews omitted experiments for protecting property other than livestock, strong inference from such studies merit tests for livestock protection. Nonetheless, given that several examples of gold standard experiments overcame the complexities of people and wild ecosystems [5, 10, 11, 12], we urge greater effort and recommend government support and accolades for the highest standards of experimentation.

## Incorporating science into conflict mitigation and conservation

Many governments have institutionalized support for livestock protection from predators and implemented various interventions at landscape scales. The European Council Directive 98/58/EC, concerning protection of animals kept for farming purposes, states that “animals not kept in buildings shall where necessary and possible be given protection from adverse weather conditions, predators and risks to their health.” The Swedish Animal Welfare Act of 1988 mandates care should be given to injured animals as soon as possible. This obligation is in practice relevant subsequent to carnivore attacks. When trained field observers confirm livestock attacks by large carnivores, they also implement rapid response interventions, such as fladry and portable electric fences, to prevent recurrent attacks [20]. In the United States, in 2013 alone, the US Department of Agriculture killed >75,000 coyotes, 320 gray wolves (*Canis lupus*), and 345 cougars (*Puma concolor*) [21]. Similarly, in some Australian states, landowners and managers are required by law to actively control dingoes (*C*. *dingo*) on their property.

Given the weak state of current evidence about effectiveness, decisions to use interventions are most likely based on subjective factors (e.g., ethics, opinions, or perceptions) or nonscientific (and thus possibly biased) evidence. For example, many people have deeply rooted perceptions that an intervention is effective or not [19]. Therefore, research, promoted by policy, is needed to validate that perceptions align with measurable and scientifically defensible outcomes [14]. This is especially crucial in cases of lethal interventions, which entail multiple drawbacks, including ethical criticisms and the potential to hasten carnivore declines and impede population recoveries.

However, scientists alone cannot transform policies for implementation. The pursuit of science-based management must be truly interdisciplinary and involve carnivore ecologists, animal husbandry scientists, social scientists, natural resource managers, ethicists, and other scholars and practitioners. Political leaders can also play a role to prioritize, coordinate, and fund partnerships across government agencies and nongovernment organizations. Because we anticipate continued debate over the standards of effectiveness, we recommend a coalition be formed to clearly distinguish standards for evaluation and experimental protocols, which would be distinct from coalitions convened to consider local factors that affect decisions. Through collaboration, scientists, managers, and policy leaders can help to protect livestock within healthy ecosystems that include carnivores. Constituents worldwide increasingly support the restoration of carnivore populations and accordingly are calling for human–carnivore coexistence and minimizing conflicts [2]. Enabling coexistence through evidence-based solutions will give the public strong confidence in methods promoted by scientists and governments, particularly when implementation is difficult or the ethics are controversial.

## Supporting information

S1 Table Methods used by authors’ reviews. Methods have been simplified for comparison. Refer to the original articles for a full account of methods used and justification for the use of these methods.
(DOCX)

S2 Table Studies included in the four reviews. (DOCX)

S1 Fig Overlap of studies included in each of the four independent reviews that evaluated evidence of functional effectiveness of interventions in reducing carnivore attacks on livestock. (TIF)
