# Supplementary material for: Carnivore conservation needs evidence-based livestock protection
Source: PLoS Biol. 2018 Sep 18;16(9):e2005577. doi: 10.1371/journal.pbio.2005577 (PMC6143182; doi:10.1371/journal.pbio.2005577)
Supplement: S1 Table — Methods have been simplified for comparison. Refer to the original articles for a full account of methods used and justification for the use of these methods. (DOCX) [file pbio.2005577.s001.docx]

**Supporting Information**

Carnivore conservation needs evidence-based livestock protection

Van Eeden et al. 2018

**S1 Table.** Methods used by authors’ reviews. Methods have been simplified for comparison. Refer to the original articles for a full account of methods used and justification for the use of these methods.

|  | **Miller et al. 2016 [6]** | **Treves et al. 2016 [7]** | **Eklund et al. 2017 [5]** | **Van Eeden et al. 2018 [8]** |
| --- | --- | --- | --- | --- |
| **Databases searched and other sources** | - Web of Science (All databases) - Carnivore Ecology and Conservation database - Snow-ball sampling | - Google scholar - Snow-ball sampling | - Zoological Record | - Web of Science (All databases) - SCOPUS - Google Scholar - European LIFE Commission Project database - Snow-ball sampling - Contacted authors and organizations |
| **Search methods and terms** | - Compound search terms included the technique (e.g., deterrent) or a specific intervention (e.g., aversive stimuli or behavior conditioning) plus 1 of 7 general keywords related to livestock depredation conflict: Human–carnivore conflict, livestock depredation, human–carnivore coexistence, mitigation, depredation management, depredation prevention, or depredation control. - Searches followed the formula: (technique or intervention) and (conflict keyword). - **Deterrents:** Aversive stimuli, Behavior conditioning, Behavior modification, Disruptive stimuli, Repellent. - **Indirect management of land or prey:** Buffer zone, Core zone, Grazing areas, Land use conflict, Wild prey, Wild ungulate. - **Predator removal:** Contraception, Lethal control, Population control, Problem animal, Retaliation, Retaliatory killing, Translocation - **Preventive husbandry:** Barrier, Grazing, Guard animal, Guard dog, Guards, Herd, Herder, Hotspot, Husbandry, Livestock breed, Penning, Sensory deterrent or repellent, Separation, Shepherd. | - Repeated searches, followed by a snowball method using the reference lists of >100 articles identified in the search. - Searched using key words: (Control, Damage, Depredation, Lethal, Non-lethal, Removal, or Livestock) AND (Predat*, Carnivor*). | - Searched using the subject descriptors: Carnivora OR Canidae OR Felidae OR Hyaenidae OR Mustelidae OR Procyonidae OR Ursidae OR Viverridae - These items were then refined using the following search string: “depredation OR stock OR poultry OR damage OR mitigation OR conflict OR control OR cull OR cow OR bull OR calf OR calves OR chicken OR hen OR ewe OR lamb OR pet OR cat OR hound OR pony OR ponies OR mule OR reindeer OR llama OR yak OR buffalo OR livestock OR cattle OR sheep OR goat OR horse OR pig OR dog OR attack OR camel OR donkey”. | Combinations of search terms from the following categories:   - **Carnivore:** Bear*, Canid*, *Canis,* Carnivore*, Cheetah*, Cougar or puma, Coyote*, *Crocuta,* Dingo*, Fox*, Hyena or hyaena, Jaguar*, Leopard*, Lion*, *Lycaeon* or *Lycaon,* Lynx*, *Panthera,* Predat*, Tiger*, *Uncia,* Wild dog*, Wildlife, Wolf, Wolves. - **Livestock:** Beef, Calf, Calves, Cattle, Chicken, Cows, Farm*, Lamb*, Poultry, Sheep, Stock. - **Impact:** Conflict, Damag*, Loss. - **Intervention:** 1080, Bait*, Chemical repellent, Compensation, Condition NEAR/2 aversion, Control, Cull, Denning, Dogging, Donkey, Farm*, Fenc*, Fladry, Guard* dog, Hunt*, Husbandry, Insurance, Livestock guard*, Livestock protect*, Llama, M-44, Management, Non$lethal, Poison, Protection collar, Range rid*, Scaring, Shoot*, Sterili*, Translocat*, Trap* - **Excluded terms:** Arthropod, Beetle*, Fish*, *flies, *fly, Hemiptera, Heteroptera, Insect*, Parasit*, Pesticide. |
| **Publications** | Peer-reviewed | Peer-reviewed | Peer-reviewed | Peer-reviewed, gray literature, and raw data |
| **Languages** | English | English and Slovenian | English | English search terms only; 3 non-English language studies were identified and included. |
| **Time period** | All years (through 2015). | All years (through 2016). | 1990-2016 | All years (through 2016). |
| **Geographic scope** | Global | North America and Europe | Global | Global |
| **Carnivore species considered** | - Large carnivores with body mass >15 kg [1]. - 28 species (all considered) | - Free- ranging, native carnivores of North America and Europe > 5 kg. - 6 species (final review) | Terrestrial mammalian large carnivore species with body mass >15 kg (Ripple, Estes (1), plus coyotes and wolverines.   - 30 species (all considered) | - Focused on large carnivores as defined by Ripple, Estes (1) but some studies considered small *and* large species (e.g. foxes, coyotes). - 11 species (final review) |
| **Definition of technique effectiveness** | Change in livestock losses or the potential for an attack (e.g., percent reduction in livestock losses or carnivore visits to a pasture) after techniques were applied. | Whether intervention will protect property owners from future losses. | Change in livestock losses (number of livestock killed, the number of livestock units attacked) or the potential for an attack (manipulation of carnivore behaviour/movement in a way that is expected to reduce exposure of livestock to carnivore predation). | - Change in livestock loss (e.g., percent loss of stock, loss of stock per period, or financial loss) and carnivore incursions into corrals or bomas. - Change in number of retaliatory killings of carnivores. - Facilitation of coexistence measured as reduction in livestock loss or retaliatory killing of carnivores. |
| **Inclusion criteria** | - Primary literature that provided numeric metrics (or values for calculating numeric metrics) of effectiveness - Reviews were omitted from analysis - Correlative studies were included. | Criteria for including studies:   1. Studies used experimental or quasi- experimental control with a design that allowed strong inference; 2. Studies occurred on working livestock operations with free- ranging, native carnivores, and 3. Studies verified livestock losses.  - Correlative studies were excluded, as well as those based only on unverified estimates of livestock loss (e.g. self- reported livestock losses or perceptions of effectiveness), and analyses in which n ≤ 4 subjects (farms or livestock herds) completed the test. | Included studies were:   - Included an empirical study of wild (i.e., not captive) carnivores; - Included a quantitative evaluation of interventions to prevent/reduce depredation of livestock (excluding apiaries); - Included a matched control to which the treatment was compared, i.e. have an experimental or quasi-experimental design. Experimental studies include a randomized case-control study design, quasi-experimental studies include a case-control study design that was not assigned randomly. - Correlative studies were excluded. - Included a description of the methods used to implement the intervention (treatment) and of a study design sufficient for replication | - Did not analyze changes in human tolerance or perceptions of carnivores; rather, included self-reported changes in livestock losses following introduction of a mitigation measure. - Studies had to be replicated with a before–after or control–impact (BACI) design. - Studies had to be field trials on livestock and at least 2 months in duration. - Excluded studies involving bait or captive carnivores - Some studies that were included did not have strict control treatments; instead compared the effects of an improvement or change in management such as electrification of fences or implementing coordinated rather than ad hoc lethal control. |
| **Data screening and harvesting** | Recorded measures of effectiveness, amount of time techniques were effective, large carnivore species involved and country where the study occurred. | - Regarding criterion (1), described in the text why any test was deemed unreliable based on selection, treatment, measurement, or reporting biases (see above). - Regarding criterion (2), defined a working livestock operation as one in which livestock, land, and predators were managed in ways characteristic of a private livestock producer. That criterion excluded tests with captive predators [18]. - Regarding criterion (3), excluded studies measuring self- reported livestock losses or perceptions of effectiveness from Table 1. - After close reading, excluded >11 studies because they did not provide reliable inference. Several tests were excluded because they were not peer-reviewed, published descriptions of all methods and results. | - 48,894 titles retrieved from primary search. - Initial manual screening of titles reduced number to 27,781. - Second manual screening (English language, depredation of domestic animals by included carnivores) left 562 publications. - Two authors read papers in full to identify correlational, quasi-experimental, or experimental studies, and identify quantitatively evaluated studies. | - Database searches returned 3146 records; 175 were added through less-structured sampling. - Mitigation methods were grouped into 5 predefined categories for the meta-analysis: lethal control, livestock guardian animals, fencing, shepherding by humans, and deterrents (e.g. aversive conditioning, repellents, and protection devices. - 40 papers describing financial incentives were discovered, including 3 that measured success, but these were not considered appropriate for comparison with other mitigation measures because the response variables were changes in farmer attitudes or retaliatory killing rather than livestock loss. |
| **Statistical units of effectiveness** | - Measures of livestock loss (e.g. number or percent livestock stock killed) - For studies reporting the effectiveness on a community of predators, reported the effectiveness for the predator community as a whole. | Livestock loss: number of livestock injured or killed by carnivores. | Mean number of animals or livestock units (e.g. herds) depredated by carnivores, or number of trespasses by carnivores. | - Measures of livestock loss, e.g. percent loss of stock, loss of stock per period, or financial loss. |
| **Data Analysis** | - Compared the effectiveness of techniques by calculating the magnitude of change between conditions before and after a technique was applied. Calculated the magnitude of change (D) as the percentage deviation from initial conditions following the formula (adapted from Jones and Schmitz (19):   *D* = ([*B – A*]/*B*) x 100  where B represents a quantitative measure of conditions (the change in livestock losses or the potential for an attack; e.g., no. of livestock killed) before the mitigation technique was applied and A represents conditions after the technique was applied.   - This metric afforded a common basis for comparing different techniques by standardizing measures of change in terms of a proportion to facilitate data integration from different studies that used different units in their response metrics. | Counted tests in various categories. Did not perform a quantitative meta-analysis of effects, because there is no standard for consistent application of treatments and because the variety of methods used even within one category (e.g. different types of traps, or breeds of livestock- guarding dogs [LGDs]) would introduce uncontrollable variation. Furthermore, tests using the silver standard offer weaker inference than those using the gold standard but to an unknown degree. | - Relative risk (or risk ratio, RR) for carnivore depredation or incursions in treatment vs. control groups for each study [20]. - RR defined as the ratio between the probability of depredation by large carnivores in the treatment group and the probability of livestock depredation by large carnivores in the control group:   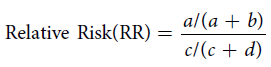where *a* is the number of depredated animals/units in the treatment group, *b* is the number of unharmed animals/ units in the treatment group, *c* is the number of depredated animals/units in the control group, and *d* is the number of unharmed animals/units in the control group.   - With no difference in the risk of depredation between treatment and control, the relative risk is 1. When RR > 1, the risk of depredation is more likely to occur in the treatment group. When RR < 1 depredation risk is higher in the control group. - For calculation of RR used the mean number of animals in treatment and control herds, as reported in the original studies (n = 1), or calculated from the reported true numbers for several herds (n = 11), as well as the number of livestock units (n = 2). Reported odds-ratios were converted to RR using an online odds ratio to risk ratio calculator [21], and Hazards Ratio were reported as in original study. Five papers did not report herd sizes; paper authors of two of these studies provided this data. | - Sample sizes, means, and standard deviations were extracted from the text, tables, or figures from each article or calculated from the data provided. - Calculated the standardized effect size as Hedges’ *d* [22] with MetaWin version 2.1 [23]. Hedges’ *d* is an estimate of the standardized mean difference between control and treatment and accounts for variation in study effort such that it is not biased by small sample size [22]. Negative values of *d* indicated the treatment successfully reduced conflict (e.g., livestock loss declined). - Data were analyzed using a random-effects model except where pooled variance was 0 (fixed-effects model used). The mean effect size per category was weighted based on variance and sample size. Total heterogeneity (*Q*_T_) was calculated for each category [23]. - Summarized data on change in carnivore killing as a proxy for tolerance because killing suggested an unwillingness to coexist. |
| **Number of studies included** | 67 | 12 | 21 | 37 |

**References**

1. Ripple WJ, Estes JA, Beschta RL, Wilmers CC, Ritchie EG, Hebblewhite M, et al. Status and ecological effects of the world’s largest carnivores. Science. 2014;343(6167):1241484. doi: 10.1126/science.1241484.

2. Chapron G, Kaczensky P, Linnell JDC, von Arx M, Huber D, Andrén H, et al. Recovery of large carnivores in Europe’s modern human-dominated landscapes. Science. 2014;346(6216):1517-9. doi: 10.1126/science.1257553.

3. Sutherland WJ, Pullin AS, Dolman PM, Knight TM. The need for evidence-based conservation. Trends in Ecology & Evolution. 2004;19(6):305-8. doi: <http://dx.doi.org/10.1016/j.tree.2004.03.018>.

4. Platt JR. Strong inference. Science. 1964;146:347-53.

5. Eklund A, López-Bao JV, Tourani M, Chapron G, Frank J. Limited evidence on the effectiveness of interventions to reduce livestock predation by large carnivores. Scientific Reports. 2017;7(1):2097.

6. Miller JRB, Stoner KJ, Cejtin MR, Meyer TK, Middleton AD, Schmidtz OJ. Effectiveness of contemporary techniques for reducing livestock depredations by large carnivores. Wildlife Society Bulletin. 2016;40(4):806-15.

7. Treves A, Krofel M, McManus J. Predator control should not be a shot in the dark. Frontiers in Ecology and the Environment. 2016;14(7):1-9. doi: 10.002/fee.1312.

8. van Eeden LM, Crowther MS, Dickman CR, Macdonald DW, Ripple WJ, Ritchie EG, et al. Managing conflict between large carnivores and livestock. Conservation Biology. 2018;32(1):26-34. doi: 10.1111/cobi.12959.

9. Iomandis JP. Why most published research findings are false. PLoS Medicine. 2005;2:e124.

10. Davidson-Nelson SJ, Gehring TM. Testing fladry as a nonlethal management tool for wolves and coyotes in Michigan. Human-Wildlife Interactions. 2010;4(1).

11. Mukherjee S. The emperor of all maladies: a biography of cancer. New York: Scribner; 2010.

12. Treves A, Wallace RB, Naughton-Treves L, Morales A. Co-managing human-wildlife conflicts: a review. Human Dimensions of Wildlife. 2006;11(6):383-96. doi: <http://dx.doi.org/10.1080/10871200600984265>.

13. Wooldridge DR. Polar bear electronic derrent and detection systems. Bears: Their Biology and Management, A Selection of Papers from the Fifth International Conference on Bear Research and Management; February 1980; Madison WI, USA: International Assocation for Bear Research and Management; 1983. p. 264-9.

14. United States Department of Agriculture. 2016 Program Data Reports: Animal and Plant Health Inspection Service, United States Department of Agriculture; 2017 [cited 2017 6 December]. Available from: <https://www.aphis.usda.gov/aphis/ourfocus/wildlifedamage/SA_Reports/SA_PDRs>.

15. López-Bao JV, Frank J, Svensson L, Åkesson M, Langefors Å. Building public trust in compensation programs through accuracy assessments of damage verification protocols. Biological Conservation. 2017;213:36-41.

16. Scasta JD, Stam B, Windh JL. Rancher-reported efficacy of lethal and non-lethal livestock predation mitigation strategies for a suite of carnivores. Scientific Reports. 2017;7:14105. doi: 10.1038/s41598-017-14462-1.

17. Santiago-Avila FJ, Cornman AM, Treves A (2018) Killing wolves to prevent predation on livestock may protect one farm but harm neighbors. PLOS ONE 13(1): e0189729. https://doi.org/10.1371/journal.pone.018972918. Jaeger MM. Selective targeting of alpha coyotes to stop sheep depredation. Sheep & Goat Research Journal. 2004;19:80-4.

19. Jones HP, Schmitz OJ. Rapid recovery of damaged ecosystems. PLoS One. 2009:4x5653.

20. Higgins JPT, Green S. Cochrane handbook for systematic reviews of Intervetions 2011 [04/04/2011]. Available from: <http://handbook.cochrane.org/front_page.htm>.

21. Kane SP. Odds Ratio to Risk Ratio calculator 2016 [04/04/2017]. Available from: <http://clincalc.com/Stats/ConvertOR.aspx>.

22. Hedges LV, Olkin I. Statistical methods for meta-analysis. Orlando, Florida: Academic Press, Inc.; 1985. 369 p.

23. Rosenberg MS, Adams DC, Gurevitch J. MetaWin: statistical software for meta-analysis. 2 ed. Sunderland, Massachusetts: Sinauer Associates, Inc.; 2000.
