# Supplementary material for: Carnivore conservation needs evidence-based livestock protection
Source: PLoS Biol. 2018 Sep 18;16(9):e2005577. doi: 10.1371/journal.pbio.2005577 (PMC6143182; doi:10.1371/journal.pbio.2005577)
Supplement: S2 Table — (DOCX) [file pbio.2005577.s002.docx]

**S2 Table.** Studies included in the four reviews.

| **Author** | **Year** | **Title** | **Country** | **Intervention** | **Carnivore** | **Livestock type** | **Duration of study** | **Journal/Source** | **Eklund et al. *[1]*** | **Miller et al. *[2]*** | **Treves et al. *[3]*** | | **van Eeden et al. *[4]*** | |
| --- | --- | --- | --- | --- | --- | --- | --- | --- | --- | --- | --- | --- | --- | --- |
| Acorn & Dorrance [5] | 1994 | An evaluation of anti-coyote electric fences | Canada | Fencing | Coyote | Sheep | 3 years | Proceedings of the 16th Vertebrate Pest Conference |  |  |  | | X | |
| Allen [6] | 2013 | Wild dog control impacts on calf wastage in extensive beef cattle enterprises | Australia | Lethal control | Dingo | Cattle | 3-4 years | Animal Production Science |  |  |  | | X | |
| Allen [7] | 2014 | More buck for less bang: reconciling competing wildlife management interests in agricultural food webs | Australia | Lethal control | Dingo | Cattle | 33 years | Food Webs |  |  |  | | X | |
| Allen & Sparkes [8] | 2001 | The effect of dingo control on sheep and beef cattle in Queensland | Australia | Lethal control | Dingo | Sheep, Cattle |  | Journal of Applied Ecology |  | X |  | |  | |
| Andelt [9] | 1992 | Effectiveness of livestock guarding dogs for reducing predation on domestic sheep | United States | Guardian animals | Coyote | Sheep | 1 year | Wildlife Society Bulletin | X |  |  | | X | |
| Andelt [10] | 1999 | Relative effectiveness of guarding-dog breeds to deter predation on domestic sheep in Colorado | United States | Guardian animals | Coyotes, dogs, mountain lions, black bears, foxes, etc. | Sheep | 8 year comparison | Wildlife Society Bulletin |  |  |  | | X | |
| Andelt & Hopper [11] | 2000 | Livestock guard dogs reduce predation on domestic sheep in Colorado | United States | Fencing, Guardian animals | American black bear | Sheep |  | Journal of Range Management |  | X |  | |  | |
| Anderson et al. [12] | 2002 | Grizzly bear-cattle interactions on two grazing allotments in northwest Wyoming | United States | Lethal control, translocation, aversive conditioning | Grizzly bear | Cattle | 2 years | Ursus |  | X |  | |  | |
| Angst [13] | 2001 | Electric fencing of fallow deer enclosures in Switzerland - a predator proof method | Switzerland | Fencing | Lynx | Deer | 4 years | Carnivore Damage Prevention News |  |  |  | | X | |
| Angst et al. [14] | 2002 | Übergriffe von Luchsen auf Kleinvieh und Gehegetiere in der Schweiz. Teil II: Massnahmen zum Schutz von Nutztieren | Switzerland | Shepherds | Lynx | Sheep, Goats, Deer | 8 years | Report: KORA |  |  |  | | X | |
| Athreya et al. [15] | 2010 | Translocation as a tool for mitigating conflict with leopards in human-dominated landscapes of India | India | Translocation | Leopard | Goats, Cattle |  | Conservation Biology |  | X |  | |  | |
| Azevedo & Murray [16] | 2007 | Evaluation of potential factors predisposing livestock to predation by jaguars | Brazil | Zoning, Land-use | Jaguar, Puma | Cattle, Water Buffalo, Goats, Fowl, Dogs, Cats | 3 years | Journal of Wildlife Management |  | X |  | |  | |
| Bagchi & Mishra [17] | 2006 | Living with large carnivores: predation on livestock by the snow leopard (*Uncia uncia*) | India | Zoning, Land-use | Snow leopard | Yak, Cattle, Cattle–yak hybrid, Horse, Donkey, Sheep, Goat | 2 years | Journal of Zoology |  | X |  | |  | |
| Bauer et al. [18] | 2015 | Financial compensation for damage to livestock by lions on community rangelands in Kenya | Kenya | Financial Incentives | Lion | Cattle, Sheep, Goats, Donkeys | 12 years | Oryx |  |  |  | | X | |
| Bauer et al. [19] | 2010 | Assessment and mitigation of human-lion conflict in West and Central Africa | Benin, Cameroon | Enclosure | Hyena, Lion | Cattle, Sheep, Goat | 2 years | Mammalia | X | X |  | |  | |
| Beckmann et al. [20] | 2004 | Evaluation of deterrent techniques and dogs to alter behavior or "nuisance" black bears | United States | Deterrents | American black bear |  | 5 years | Wildlife Society Bulletin |  | X |  | |  | |
| Bjorge & Gunson [21] | 1985 | Evaluation of wolf control to reduce cattle predation in Alberta | Canada | Lethal control | Wolf | Cattle | 6 years | Journal of Range Management |  |  |  | | X | |
| Blejwas et al. [22] | 2002 | The effectiveness of selective removal of breeding coyotes in reducing sheep predation | United States | Lethal control | Coyote | Sheep | 2.8 | Journal of Wildlife Management | X |  |  | |  | |
| Bradley & Pletscher [23] | 2005 | Assessing factors related to wolf depredation of cattle in fenced pastures in Montana & Idaho | United States | Preventive husbandry | Wolf | Cattle | 8 years | Wildlife Society Bulletin |  | X |  | |  | |
| Bradley et al. [24] | 2004 | An evaluation of wolf-livestock conflicts and management in the Northwestern United States (MS thesis) | United States |  | Wolf |  |  | MSc Thesis: University of Montana |  |  | X | |  | |
| Bradley et al. [25] | 2005 | Evaluating wolf translocation as a nonlethal method to reduce livestock conflicts in the northwestern United States | United States | Translocation | Wolf | Unclear | 13 years | Conservation Biology |  | X |  | |  | |
| Bradley et al. [26] | 2015 | Effects of wolf removal on livestock depredation recurrence and wolf recovery in Montana, Idaho, and Wyoming | United States | Lethal control, Translocation | Wolf | Sheep, Cattle, Other | 1850 days | Journal of Wildlife Management | X |  |  | |  | |
| Breck et al. [27] | 2006 | A shocking device for protection of concentrated food sources from black bears | United States | Deterrents | Wolf | Sheep | 3 years | Wildlife Society Bulletin |  | X |  | |  | |
| Breck et al. [28] | 2002 | Non-lethal radio activated guard for deterring wolf depredation in Idaho: summary and call for research | United States | Deterrents | American black bear |  |  | Proceedings of the 20th Vertebrate Pest Conference |  | X |  | |  | |
| Breck et al. [29] | 2011 | Domestic calf mortality and producer detection rates in the Mexican wolf recovery area: implications for livestock management and carnivore compensation schemes | United States | Calving time | Mexican wolf | Cattle | 4 years | Biological Conservation |  | X |  | |  | |
| Bromley & Gese [30] | 2001 | Surgical sterilization as a method of reducing coyote predation on domestic sheep | United States | Sterilization | Coyote | Lambs | 5-23 days | Journal of Wildlife Management | X |  |  | |  | |
| Ciucci & Boitani [31] | 1998 | Wolf and dog depredation on livestock in central Italy | Italy | Fencing | Wolf |  |  | Wildlife Society Bulletin |  | X |  | |  | |
| Conner et al. [32] | 1998 | Effect of coyote removal on sheep depredation in northern California | United States | Lethal control | Coyote | Sheep |  | Journal of Wildlife Management |  |  | X | |  | |
| Davidson-Nelson & Gehring [33] | 2010 | Testing fladry as a nonlethal management tool for wolves and coyotes in Michigan | United States | Fladry | Wolves and Coyotes | Sheep, Cattle | 75 days | Human-Wildlife Interactions | X |  | X | |  | |
| deCalesta & Cropsey [34] | 1978 | Field test of a coyote-proof fence | United States | Fencing | Coyote | Sheep | 1 year | Wildlife Society Bulletin |  |  |  | | X | |
| Dorrance & Bourne [35] | 1980 | An evaluation of anti-coyote electric fencing | Canada | Fencing | Coyote | Sheep | 5 years | Journal of Range Management |  |  |  | | X | |
| Edgar et al. [36] | 2007 | Efficacy of an ultrasonic device as a deterrent to dingoes (*Canis lupus dingo*): a preliminary investigation | Australia | Deterrents | Dingo | None (captive experiments) | | Journal of Ethology |  | X |  | |  | |
| Ellins [37] | 2005 | Conditioned prey aversions (book chapter in Living with Coyotes) | United States | Deterrents | Coyote | Sheep | 2 years | Book Chapter: Living with Coyotes |  |  |  | | X | |
| Espuno et al. [38] | 2004 | Heterogeneous response to preventive sheep husbandry during wolf recolonization of the French Alps | France | Guardian dogs and/or night time corralling | Wolf | Sheep | 7 years | Wildlife Society B |  | X | X | |  | |
| Gehring et al. [39] | 2010 | Utility of livestock-protection dogs for deterring wildlife from cattle farms | United States | LGDs | Wolf | Cattle | Multiple years | Wildlife Research | X | X | X | |  | |
| Gehring et al. [40] | 2006 | Are viable non-lethal management tools available for reducing wolf-human conflict? Preliminary results from field experiments | United States | Deterrents, Fladry | Wolf | Sheep, Cattle | 2 years | Proceedings of the 22nd Vertebrate Pest Conference |  | X |  | |  | |
| Goodrich & Miquelle [41] | 2005 | Translocation of problem Amur tigers *Panthera tigris altaica* to alleviate tiger-human conflicts | Russia | Translocation | Tiger |  | Multiple years | Oryx |  | X |  | |  | |
| Gula [42] | 2008 | Wolf depredation on domestic animals in the Polish Carpathian Mountains | Poland | None: correlative | Wolf | Sheep | 6 years | Journal of Wildlife Management |  | X |  | |  | |
| Gusset et al. [43] | 2009 | Human-wildlife conflict in northern Botswana: livestock predation by endangered African wild dog | Botswana | Enclosures | African Wild Dog |  |  | Oryx |  | X |  | |  | |
| Gustavson et al. [44] | 1982 | A 3-year evaluation of taste aversion coyote control in Saskatchewan | Canada | Deterrents | Coyote | Sheep | 4 years | Journal of Range Management |  |  |  | | X | |
| Hansen & Smith [45] | 1999 | Livestock-guarding dogs in Norway Part II: different working regimes | Norway | Guardian animals | Brown bear | Sheep | 3 months | Journal of Range Management |  | X |  | |  | |
| Harper et al. [46] | 2008 | Effectiveness of lethal, directed wolf-depredation control in Minnesota | United States | Lethal control | Wolf | Cattle, Sheep, Turkey | 20 years | Journal of Wildlife Management | X | X |  | |  | |
| Hawley et al. [47] | 2009 | Assessment of shock collars as nonlethal management for wolves in Wisconsin | United States | Deterrents | Wolf | Bait | 28 days | Journal of Wildlife Management | X | X |  | |  | |
| Hazzah et al. [48] | 2014 | Efficacy of two lion conservation programs in Maasailand, Kenya | Kenya | Financial incentives and other | Lions | Cattle | 11 years | Conservation Biology |  |  |  | | X | |
| Herfindal et al. [49] | 2005 | Does recreational hunting of lynx reduce depredation losses of domestic sheep? | Norway | Lethal control | Lynx | Sheep | 6 years | Journal of Wildlife Management |  | X | X | |  | |
| Herrero & Higgins [50] | 1998 | Field use of capsicum spray as a bear deterrent | United States and Canada | Deterrents | American black bear, brown bear | | 10 years | Ursus |  | X |  | |  | |
| Huygens & Hayashi [51] | 1999 | Using electric bear fences to reduce Asiatic black bear depredation in Nagano prefecture, central Japan | Japan | Fencing | Asiatic black bear |  | 5 years | Wildlife Society Bulletin |  | X |  | |  | |
| Huygens et al. [52] | 2004 | Relationships between Asiatic black bear kills and depredation costs in Nagano prefecture, Japan | Japan | Lethal control | Asiatic black bear |  |  | Ursus |  | X |  | |  | |
| Iliopolous et al. [53] | 2009 | Wolf depredation on livestock in central Greece | Greece | Shepherds | Wolf | Sheep, Goats | 21 months | Acta Theriologica | X | X |  | |  | |
| Jankovsky et al. [54] | 1974 | Field trials of coyote repellents in western Colorado | United States | Deterrents | Coyote | Sheep | 4 months | Proceedings of the Western Section of the American Society of Animal Science |  |  | |  | | X |
| Jelinski et al. [55] | 1983 | Coyote predation on sheep, and control by aversive condition in Saskatchewan | Canada | Deterrents | Coyote | Sheep | 2 years | Journal of Range Management |  |  |  | | X | |
| Karanth et al. [56] | 2013 | Patterns of human-wildlife conflicts and compensation: insights from Western Ghats protected areas | India | Night watching, fencing, scare devices, guard animals | Tiger, Leopard, Fox |  | 2 years | Biological Conservation |  | X |  | |  | |
| Kavcic et al. [57] | 2013 | Supplemental feeding with carrion is not reducing brown bear depredations on sheep in Slovenia | Slovenia | Supplementary feeding | Brown bear |  |  | Ursus |  |  | X | |  | |
| Kolowski & Holecamp [58] | 2006 | Spatial, temporal, and physical characteristics of livestock depredation by large carnivores along a Kenyan reserve border | Kenya | Enclosure | Hyena, Leopard | Goat, Sheep | 14 months | Biological Conservation | X | X |  | |  | |
| Krofel et al. [59] | 2011 | Effectiveness of wolf (*Canis lupus*) culling as a measure to reduce livestock depredations | Slovenia | Lethal control | Wolf |  |  | Acta Silvae et Ligni |  |  | X | |  | |
| Krogstad et al. [60] | 2000 | Protective measures against depredation on sheep: shepherding and use of livestock guardian dogs in Lierne. Final report - 2000. | Norway | Guardian animals | Lynx & wolverine | Sheep | 4 years | Report: NINA |  |  |  | | X | |
| Lance et al. [61] | 2009 | Biological, technical, and social aspects of applying electrified fladry for livestock protection from wolves (*Canis lupus*) | United States | Fladry | Wolf | Cattle | 49 days | Wildlife Research | X | X |  | |  | |
| Landa et al. [62] | 1999 | Factors associated with wolverine *Gulo gulo* predation on domestic cheep | Norway | Change livestock | Wolverine | Sheep | 3 years | Journal of Applied Ecology | X |  |  | |  | |
| Landriault et al | 2009 | Age, sex, and relocation distance as predictors of return for relocated nuisance black bears *Ursus americanus* in Ontario, Canada | Canada | Translocation | American black bear |  | 15 years | Wildlife Biology |  | X |  | |  | |
| Landry & Raydelet [63] | 2010 | Efficacité des chiens de protection contre la prédation du lynx dans le Massif jurassien: Présentation préliminaire des résultats de l’enquête de terrain | France | Guardian animals | Lynx | Sheep | 23 years | Report: Pôle Grands Prédateurs |  |  |  | | X | |
| Leigh [64] | 2007 | Effects of aversive conditioning behavior of nuisance Louisiana black bears (Thesis) | United States | Deterrents | American black bear |  |  | Louisiana State University |  | X |  | |  | |
| Lichtenfeld et al. [65] | 2015 | Evidence-based conservation: predator-proof bomas protect livestock and lions | Tanzania | Fencing (bomas and fortified bomas) | Lions | Cattle, Shoats, Donkeys | 10 years (9296 boma months) | Biodiversity & Conservation | X |  |  | | X | |
| Linhart et al. [66] | 1982 | Electric fencing reduces coyote predation on pastured sheep | United States | Fencing | Coyote | Sheep | Average 65.67 nights | Journal of Range Management |  |  |  | | X | |
| Linhart et al. [67] | 1984 | Efficacy of light and sound stimuli for reducing coyote predation upon pastured sheep | United States | Deterrents | Coyote | Sheep | 2 years | Protection Ecology |  |  |  | | X | |
| Linhart et al. [68] | 1992 | Electronic frightening devices for reducing coyote predation on domestic sheep: efficacy under range conditions and operational use | United States | Deterrents | Coyote | Sheep | 5 years | Proceedings of the 15th Vertebrate Pest Conference |  |  |  | | X | |
| Maclennan et al. [69] | 2009 | Evaluation of a compensation scheme to bring about pastoral tolerance of lions | Kenya | Financial incentives | Lions | Cattle, Donkeys, Sheep, Goats | 6 years | Biological Conservation |  |  |  | | X | |
| Mahoney & Charry [70] | 2007 | The use of alpacas as new-born lamb protectors to minimise fox predation | Australia | Guardian animals | Dingo and fox | Lambs | 14 weeks | Extension Farming Systems Journal |  |  |  | | X | |
| Marker et al. [71] | 2005 | Survivorship and causes of mortality for livestock-guarding dogs on Namibian Rangeland | Namibia | Guardian animals | Cheetah |  | 7 years | Rangeland Ecology and Management |  | X |  | |  | |
| Martin and O'Brien [72] | 2000 | The use of bone oil (Renardine) as a coyote repellent on sheep farms in Ontario | Canada | Deterrents | Coyote | Sheep | 4-5 years | Proceedings of the 19th Vertebrate Pest Conference |  |  |  | | X | |
| Mazzolli et al. [73] | 2002 | Mountain lion depredation in southern Brazil | Brazil | Night enclosure | Puma | Sheep, Swine | 3 years | Biological Conservation | X | X |  | |  | |
| McManus et al. [74] | 2014 | Dead or alive? Comparing costs and benefits of lethal and non-lethal human-wildlife conflict mitigation on livestock farms | South Africa | Lethal control | Black-backed jackal, caracal, leopard | | 3 years | Oryx |  | X |  | |  | |
| Meadows & Knowlton [75] | 2000 | Efficacy of guard llamas to reduce canine predation on domestic sheep | United States | Guardian animals | Coyote | Sheep | 80 weeks | Wildlife Society Bulletin |  |  |  | | X | |
| Mech et al. [76] | 2000 | Assessing factors that may predispose Minnesota farms to wolf depredations on cattle | United States | Preventive husbandry | Wolf | Cattle |  | Wildlife Society Bulletin |  | X |  | |  | |
| Michalski et al. [77] | 2006 | Human-wildlife conflicts in a fragmented Amazonian forest landscape: determinants of large felid depredationon livestock | Brazil | Preventive husbandry | Jaguars & Pumas | Cattle | 4 years | Animal Conservation |  | X |  | |  | |
| Miller [78] | 1987 | Field tests of potential polar bear repellents | Canada | Deterrents | Polar Bear |  | 2 months | International Conference on Bear Restoration |  | X |  | |  | |
| Mitchell et al. [79] | 2004 | Coyote depredation management: current methods and research needs | United States and Canada | | Coyote |  |  | Wildlife Society Bulletin |  |  | X | |  | |
| Musiani et al. [80] | 2003 | Wolf depredation trends and the use of fladry barriers to protect livestock in western North America | Canada | Fladry | Wolf | Cattle/bait | 60 days | Conservation Biology | X | X |  | |  | |
| Nass & Theade [81] | 1988 | Electric fences for reducing sheep losses to predators | United States | Fencing | Coyotes and dogs | Sheep | Average 4.1 years treatment | Journal of Range Management |  |  |  | | X | |
| National Project Steering Committee [82] | 2014 | National Wild Dog Action Plan - Brindabella Wee Jasper case study | Australia | Lethal control | Dingo | Sheep | 20 years | Report: National Wild Dog Action Plan |  |  |  | | X | |
| Obbard et al. [83] | 2014 | Relationships among food availability, harvest, and human-bear conflict at landscape scales in Ontario, Canada | Canada |  | American black bear |  |  | Ursus |  |  | X | |  | |
| Odden et al. [84] | 2008 | Vulnerability of domestic sheep to lynx depredation in relation to roe deer density | Norway | Wild prey availability | Lynx | Sheep | 9 years | Journal of Wildlife Management |  | X |  | |  | |
| Odden et al. [85] | 2013 | Density of wild prey modulates lynx kill rates on free-ranging domestic sheep | Norway | Wild prey availability | Lynx | Sheep | 16 years | PLoS ONE |  | X |  | |  | |
| Ogada et al. [86] | 2003 | Limiting depredation by African carnivores: the role of livestock husbandry | Kenya | Husbandry | Lions, leopards, cheetahs, spotted hyenas | Cattle, Sheep, Goats | 1 year | Conservation Biology |  | X |  | |  | |
| Otstavel et al. [87] | 2009 | The first experience of livestock guarding dogs preventing large carnivore damages in Finland | Finland | Guardian animals | Lynx, Brown bear, Wolf | Sheep, Cattle, Poultry, Horses, Alpaca, Donkey |  | Estonian Journal of Ecology |  | X |  | |  | |
| Palmer et al. [88] | 2010 | Replication of a 1970s study on domestic sheep losses to predators on Utah's summer rangelands | United States | Guardian animals, Shepherds | Coyotes, cougars, black bears | Sheep | 4 months | Rangeland Ecology and Management | X |  |  | | X | |
| Peebles et al. [89] | 2013 | Effects of remedial sport hunting on cougar complaints and livestock depredations | United States | Lethal control | Cougar |  |  | PLoS ONE |  |  | X | |  | |
| Rigg et al. [90] | 2011 | Mitigating carnivore-livestock conflict in Europe: lessons from Slovakia | Slovakia | Night enclosure, Guardian animals | Brown bear, wolf | Sheep | 3 years | Oryx | X | X |  | |  | |
| Rossler et al. [91] | 2012 | Shock collars as a site-aversive conditioning tool for wolves | United States | Deterrents | Wolf | Cattle, Sheep, Horse | 2 years | Wildlife Society Bulletin |  | X |  | |  | |
| Rust et al. [92] | 2013 | Perceived efficacy of livestock-guarding dogs in South Africa: implications for cheetah conservation | South Africa | Guardian animals | Cheetah | Sheep, Goats, Cattle | 2 years and 2 months | Wildlife Society Bulletin |  |  |  | | X | |
| Sagør et al. [93] | 1997 | Compatibility of brown bear *Ursus arctos* and free-ranging sheep in Norway | Norway | Lethal control | Brown bear | Sheep | 12 years | Biological Conservation |  | X | X | |  | |
| Salvatori & Mertens [94] | 2012 | Damage prevention methods in Europe: experiences from LIFE nature projects | Italy, Spain, Portugal, France, Croatia | Guardian animals, Fencing | Brown bear and wolf | Bulls, cattle, goats, sheep, bee-hives, orchards |  | Hystrix |  | X |  | |  | |
| Sampson & Brohn [95] | 1955 | Missouri's program of extension predator control | United States | Lethal control | Coyotes | Not specified (but sponsored by Missouri Sheep and Wool Growers Association) | 8 years | The Journal of Wildlife Management |  |  |  | | X | |
| Schultz et al. [96] | 2005 | Experimental use of dog-training shock collars to deter depredation by gray wolves | United States | Deterrents | Wolf |  | 4 years | Wildlife Society Bulletin |  | X |  | |  | |
| Shivik et al. [97] | 2003 | Nonlethal techniques for managing predation: primary and secondary repellents | United States | Deterrents, Fladry | Wolf | None (baits) | 2 months | Conservation Biology |  | X |  | |  | |
| Stahl et al. [98] | 2001 | The effect of removing lynx in reducing attacks on sheep in the French Jura Mountains | France | Lethal control | Lynx | Sheep | Average 7.22 months | Biological Conservation |  | X |  | | X | |
| Stahl et al. [99] | 2002 | Factors effecting lynx predation on sheep in the French Jura | France | Land-use, wild prey | Lynx | Sheep | 4 years | Journal of Applied Ecology |  | X |  | |  | |
| Stander [100] | 1990 | A suggested management strategy for stock-raiding lions in Namibia | Namibia | Translocation, Lethal Control | Lions | Cattle | 3 years | South African Journal of Wildlife Research |  | X |  | |  | |
| Suryawanshi et al. [101] | 2013 | People, predators and perceptions: patterns of livestock depredation by snow leopards and wolves. |  | Land-use | Snow leopard, wolf | Yak, Horse | 5 years | Journal of Applied Ecology |  | X |  | |  | |
| Swanson & Scott [102] | 1973 | Livestock protectors for sheep predator control | United States | Deterrents | Coyotes | Sheep | 3 years | Proceedings of the Western Section of the American Society of Animal Science |  |  | |  | | X |
| Treves et al. [103] | 2011 | Forecasting environmental hazards and the application of risk maps to predator attacks on livestock | United States | Land-use | Wolf | Cattle | 7 years | Bioscience |  | X |  | |  | |
| Tumenta et al. [104] | 2013 | Livestock depredation and mitigation methods practised by resident and nomadic pastoralists around Waza National Park, Cameroon | Cameroon | Night enclosure | Lions | Cattle, Sheep, Goat |  | Oryx |  | X |  | |  | |
| Valeix et al. [105] | 2012 | Behavioural adjustments of a large carnivore to access secondary prey in a human-dominated landscape | Botswana | Wild prey availability | Lions | Cattle | 2 years | Journal of Applied Ecology |  | X |  | |  | |
| van Bommel [106] | 2013 | Guardian dogs for livestock protection in Australia | Australia | Guardian animals | Dingo | Goats, calves, lambs and poultry | Varied (up to 30 years) | Thesis: The University of Tasmania |  |  |  | | X | |
| van Bommel & Johnson [107] | 2012 | Good dog! Using livestock guardian dogs to protect livestock from predators in Australia's extensive grazing systems | Australia | Guardian animals | Dingo | Sheep, Goat | 7 months | Wildlife Research |  | X |  | |  | |
| van Bommel et al. [108] | 2007 | Factors affecting livestock predation by lions in Cameroon | Cameroon | Preventive husbandry | Lions | Cattle, Sheep, Goat | 2 months | African Journal of Ecology |  | X |  | |  | |
| van Liere et al. [109] | 2013 | Farm characteristics in Slovene wolf habitat related to attacks on sheep | Slovenia | Night enclosure | wolf | Sheep | 5 months | Applied Animal Behaviour Science |  | X |  | |  | |
| Wagner & Conover [110] | 1999 | Effect of preventive coyote hunting on sheep losses to coyote predation | United States | Lethal control | Coyote | Lambs | 3-6 months | The Journal of Wildlife Management | X |  |  | | X | |
| Walking for Lions [111] | 2016 | Quarterly Report | Botswana | Deterrents | Lions | Includes cattle | 2 months | Report: Walking for Lions |  |  |  | | X | |
| Wilson et al. [112] | 2005 | Natural landscape features, human-related attractants, and conflict hotspots: a spatial analysis of human-grizzly bear conflicts | United States | Calving, fencing | Grizzly bear | Cattle, Sheep, Beehives | 15 years | Ursus |  | X |  | |  | |
| Woodroffe et al. [113] | 2007 | Livestock husbandry as a tool for carnivore conservation in Africa's community rangelands: a case-control study | Kenya | Shepherds, Guardian animals, Scarecrows, Fencing | Lion, Leopard, Hyena | Cattle, sheep and goats, camels, donkeys | 4.5 years | Biodiversity & Conservation | X | X |  | |  | |
| Woodroffe et al. [114] | 2005 | Livestock predation by endangered African wild dogs (*Lycaeon pictus*) in northern Kenya | Kenya | Land-use, preventive husbandry | African Wild Dog | Goat, Sheep, Cattle | 3 years | Biological Conservation |  | X |  | |  | |
| Wooldridge [115] | 1983 | Polar bear electronic deterrent and detection systems | Canada | Deterrent | Polar bear |  | 4 years | Bears: Their Biology & Management |  | X |  | |  | |
| Zarco-González & Monroy-Vilchis [116] | 2014 | Effectiveness of low-cost deterrents in decreasing livestock predation by felids: a case study in Central Mexico | Mexico | Deterrents | Puma and jaguar | Cattle, goats | 2 months | Animal Conservation | X | X |  | | X | |

**References**

1. Eklund A, López-Bao JV, Tourani M, Chapron G, Frank J. Limited evidence on the effectiveness of interventions to reduce livestock predation by large carnivores. Scientific Reports. 2017;7(1):2097. doi: 10.1038/s41598-017-02323-w.

2. Miller JRB, Stoner KJ, Cejtin MR, Meyer TK, Middleton AD, Schmidtz OJ. Effectiveness of contemporary techniques for reducing livestock depredations by large carnivores. Wildlife Society Bulletin. 2016;40(4):806-15. doi: 10.1002/wsb.720

3. Treves A, Krofel M, McManus J. Predator control should not be a shot in the dark. Frontiers in Ecology and the Environment. 2016;14(7):1-9. doi: 10.002/fee.1312.

4. van Eeden LM, Crowther MS, Dickman CR, Macdonald DW, Ripple WJ, Ritchie EG, et al. Managing conflict between large carnivores and livestock. Conservation Biology. 2018;32(1):26-34. doi: 10.1111/cobi.12959.

5. Acorn RC, Dorrance MJ. An evaluation of anti-coyote electric fences. Proceedings of the Sixteenth Vertebrate Pest Conference1994. p. 45-50.

6. Allen LR. Wild dog control impacts on calf wastage in extensive beef cattle enterprises. Animal Production Science. 2013;54(2):214-20. doi: 10.1071/AN12356.

7. Allen BL. More buck for less bang: Reconciling competing wildlife management interests in agricultural food webs. Food Webs. 2015;2:1-9. doi: 10.1016/j.fooweb.2014.12.001.

8. Allen LR, Sparkes EC. The effect of dingo control on sheep and beef cattle in Queensland. Journal of Applied Ecology. 2001;38(1):76-87. doi: 10.1046/j.1365-2664.2001.00569.x.

9. Andelt WF. Effectiveness of livestock guarding dogs for reducing predation on domestic sheep. Wildlife Society Bulletin (1973-2006). 1992;20(1):55-62.

10. Andelt WF. Relative effectiveness of guarding-dog breeds to deter predation on domestic sheep in Colorado. Wildlife Society Bulletin (1973-2006). 1999;27(3):706-14.

11. Andelt WF, Hopper SN. Livestock guard dogs reduce predation on domestic sheep in Colorado. Journal of Range Management. 2000;53(3):259-67. doi: 10.2307/4003429.

12. Anderson CR, Ternent MA, Moody DS. Grizzly bear-cattle interactions on two grazing allotments in Northwest Wyoming. Ursus. 2002;13:247-56.

13. Angst C. Electric fencing of fallow deer enclosures in Switzerland - predator proof method. Carnivore Damage Prevention News. 2001;3:8-9.

14. Angst C, Hagen S, Breitenmoser U. Übergriffe von Luchsen auf Kleinvieh und Gehegetiere in der Schweiz. Teil II: Massnahmen zum Schutz von Nutztieren. Muri, Switzerland: KORA; 2002. p. 65.

15. Athreya V, Odden M, Linnell JDC, Karanth KU. Translocation as a tool for mitigating conflict with leopards in human-dominated landscapes of India. Conservation Biology. 2010;25(1):133-41. doi: 10.1111/j.1523-1739.2010.01599.x.

16. Azevedo FCCD, Murray DL. Evaluation of potential factors predisposing livestock to predation by jaguars. Journal of Wildlife Management. 2007;71(7):2379-86. doi: 10.2193/2006-520.

17. Bagchi S, Mishra C. Living with large carnivores: predation on livestock by the snow leopard (*Uncia uncia*). Journal of Zoology. 2006;268(3):217-24. doi: doi:10.1111/j.1469-7998.2005.00030.x.

18. Bauer H, Müller L, Van Der Goes D, Sillero-Zubiri C. Financial compensation for damage to livestock by lions *Panthera leo* on community rangelands in Kenya. ORYX. 2015. doi: 10.1017/S003060531500068X.

19. Bauer H, de Iongh H, Sogbohossou E. Assessment and mitigation of human-lion conflict in West and Central Africa. Mammalia. 2010;74:363-7. doi: 10.1515/MAMM.2010.048.

20. Beckmann JP, Lackey CW, Berger J. Evaluation of deterrent techniques and dogs to alter behavior of “nuisance” black bears. Wildlife Society Bulletin. 2004;32(4):1141-6. doi: doi:10.2193/0091-7648(2004)032[1141:EODTAD]2.0.CO;2.

21. Bjorge RR, Gunson JR. Evaluation of wolf control to reduce cattle predation in Alberta. Journal of Range Management. 1985;(38):6.

22. Blejwas KM, Sacks BN, Jaeger MM, McCullough DR. The effectiveness of selective removal of breeding coyotes in reducing sheep predation. The Journal of Wildlife Management. 2002;66(2):451-62. doi: 10.2307/3803178.

23. Bradley EH, Pletscher DH. Assessing factors related to wolf depredation of cattle in fenced pastures in Montana and Idaho. Wildlife Society Bulletin. 2005;33(4):1256-65. doi: 10.2193/0091-7648(2005)33[1256:AFRTWD]2.0.CO;2.

24. Bradley EH. An evaluation of wolf-livestock conflicts and management in the Northwestern United States [Masters Thesis]. Missoula: The University of Montana; 2004.

25. Bradley EH, Pletscher DH, Bangs EE, Kunkel KE, Smith DW, Mack CM, et al. Evaluating wolf translocation as a nonlethal method to reduce livestock conflicts in the Northwestern United States. Conservation Biology. 2005;19(5):1498-508. doi: 10.1111/j.1523-1739.2005.00102.x.

26. Bradley EH, Robinson HS, Bangs EE, Kunkel K, Jimenez MD, Gude JA, et al. Effects of wolf removal on livestock depredation recurrence and wolf recovery in Montana, Idaho, and Wyoming. The Journal of Wildlife Management. 2015;79(8):1337-46. doi: 10.1002/jwmg.948.

27. Breck SW, Lance N, Callahan P. A shocking device for protection of concentrated food sources from black bears. Wildlife Society Bulletin. 2006;34(1):23-6. doi:10.2193/0091-7648(2006)34[23:ASDFPO]2.0.CO;2.

28. Breck SW, Williamson R, Niemeyer C, Shivik JA, editors. Non-lethal radio activated guard for deterring wolf depredation in Idaho: summary and call for research. Proceedings of the Vertebrate Pest Conference; 2002; Davis: University of California.

29. Breck SW, Kluever BM, Panasci M, Oakleaf J, Johnson T, Ballard W, et al. Domestic calf mortality and producer detection rates in the Mexican wolf recovery area: Implications for livestock management and carnivore compensation schemes. Biological Conservation. 2011;144(2):930-6. doi: 10.1016/j.biocon.2010.12.014.

30. Bromley C, Gese EM. Surgical sterilization as a method of reducing coyote predation on domestic sheep. Journal of Wildlife Management. 2001;65(3):510-9.

31. Ciucci P, Boitani L. Wolf and dog depredation on livestock in central Italy. Wildlife Society Bulletin (1973-2006). 1998;26(3):504-14.

32. Conner MM, Jaeger MM, Weller TJ, McCullough DR. Effect of coyote removal on sheep depredation in northern California. Journal of Wildlife Management. 1998;62(2):690-9. doi: 10.2307/3802345.

33. Davidson-Nelson SJ, Gehring TM. Testing fladry as a nonlethal management tool for wolves and coyotes in Michigan. Human-Wildlife Interactions. 2010;4(1):87-94.

34. deCalesta DS, Cropsey MG. Field test of a coyote-proof fence. Wildlife Society Bulletin. 1978;6(4):256-9.

35. Dorrance MJ, Bourne J. An evaluation of anti-coyote electric fencing. Journal of Range Management. 1980;33:385-7.

36. Edgar JP, Appleby RG, Jones DN. Efficacy of an ultrasonic device as a deterrent to dingoes (*Canis lupus dingo*): a preliminary investigation. Journal of Ethology. 2007;25(2):209-13. doi: 10.1007/s10164-006-0004-1.

37. Ellins SR. Living with coyotes: managing predators humanely using food aversion conditioning. Austin: University of Texas Press; 2005. 175 p.

38. Espuno N, Lequette B, Poulle M-L, Migot P, Lebreton J-D. Heterogeneous response to preventive sheep husbandry during wolf recolonization of the French Alps. Wildlife Society Bulletin. 2004;32(4):1195-208. doi: 10.2193/0091-7648(2004)032[1195:HRTPSH]2.0.CO;2.

39. Gehring TM, VerCauteren KC, Provost ML, Cellar AC. Utility of livestock-protection dogs for deterring wildlife from cattle farms. Wildlife Research. 2010;37(8):715-21. doi: 10.1071/wr10023.

40. Gehring TM, Hawley JE, Davidson SJ, Rossler ST, Cellar AC, Schultz RN, et al. Are viable non-lethal management tools available for reducing wolf-human conflict? Preliminary results from field experiments. Proceedings of the 22nd Vertebrate Pest Conference. 2006:2-6.

41. Goodrich JM, Miquelle DG. Translocation of problem Amur tigers *Panthera tigris altaica* to alleviate tiger-human conflicts. Oryx. 2005;39(04):454-7. doi: 10.1017/S0030605305001146.

42. Gula R. Wolf depredation on domestic animals in the Polish Carpathian Mountains. Journal of Wildlife Management. 2008;72(1):283-9. doi: 10.2193/2006-368.

43. Gusset M, Swarner MJ, Mponwane L, Keletile K, McNutt JW. Human-wildlife conflict in northern Botswana: livestock predation by Endangered African wild dog *Lycaon pictus* and other carnivores. Oryx. 2009;43(1):67-72. doi: 10.1017/s0030605308990475.

44. Gustavson CR, Jowsey JR, Milligan DN. A 3-year evaluation of taste aversion coyote control in Saskatchewan. Journal of Range Management. 1982;35(1):57-9.

45. Hansen I, Smith ME. Livestock-guarding dogs in Norway, Part II: different working regimes. Journal of Range Management. 1999;52:312-6. doi: 10.2307/4003539.

46. Harper EK, Paul WJ, Mech LD, Weisberg S. Effectiveness of lethal, directed wolf-depredation control in Minnesota. Journal of Wildlife Management. 2008;72(3):778-84. doi: 10.2193/2007-273.

47. Hawley JE, Gehring TM, Schultz RN, Rossler ST, Wydeven AP. Assessment of shock collars as nonlethal management for wolves in Wisconsin. Journal of Wildlife Management. 2009;73(4):518-25. doi: 10.2193/2007-066.

48. Hazzah L, Dolrenry S, Naughton L, Edwards CT, Mwebi O, Kearney F, et al. Efficacy of two lion conservation programs in Maasailand, Kenya. Conservation Biology. 2014;28(3):851-60. doi: 10.1111/cobi.12244.

49. Herfindal I, Linnell JDC, Moa PF, Odden J, Austmo LB, Andersen R. Does recreational hunting of lynx reduce depredation losses of domestic sheep? Journal of Wildlife Management. 2005;69(3):1034-42. doi: 10.2193/0022-541X(2005)069[1034:DRHOLR]2.0.CO;2.

50. Herrero S, Higgins A. Field use of capsicum spray as a bear deterrent. Ursus. 1998;10:533-7.

51. Huygens OC, Hayashi H. Using electric fences to reduce Asiatic black bear depredation in Nagano Prefecture, central Japan. Wildlife Society Bulletin (1973-2006). 1999;27(4):959-64.

52. Huygens OC, van Manen FT, Martorello DA, Hayashi H, Ishida J. Relationships between Asiatic black bear kills and depredation costs in Nagano Prefecture, Japan. Ursus. 2004;15(2):197-202.

53. Iliopoulos Y, Sgardelis S, Koutis V, Savaris D. Wolf depredation on livestock in central Greece. Mammal Research. 2009;54(1):11-22. doi: 10.1007/bf03193133.

54. Jankovsky MJ, Swanson VB, Cramer DA. Field trials of coyote repellents in Western Colorado. Proceedings, Western Section, American Society of Animal Science. 1974;25:74-6.

55. Jelinski DE, Rounds RC, Jowsey JR. Coyote predation on sheep, and control by aversive conditioning in Saskatchewan. Journal of Range Management. 1983;36(1):16-9. doi: 10.2307/3897972.

56. Karanth KK, Gopalaswamy AM, Prasad PK, Dasgupta S. Patterns of human–wildlife conflicts and compensation: Insights from Western Ghats protected areas. Biological Conservation. 2013;166:175-85. doi: 10.1016/j.biocon.2013.06.027.

57. Kavčič I, Adamič M, Kaczensky P, Krofel M, Jerina K. Supplemental feeding with carrion is not reducing brown bear depredations on sheep in Slovenia. Ursus. 2013;24(2):111-9. doi: 10.2192/URSUS-D-12-00031R1.1.

58. Kolowski JM, Holekamp KE. Spatial, temporal, and physical characteristics of livestock depredations by large carnivores along a Kenyan reserve border. Biological Conservation. 2006;128:529-41. doi: 10.1016/j.biocon.2005.10.021.

59. Krofel M, Černe R, Jerina K. Effectivness of wolf (*Canis lupus*) culling as a measure to reduce livestock depredations. Zbornik Gozdarstva in Lesarstva. 2011;(95).

60. Krogstad S, Christiansen F, Smith ME, Røste OC, Aanesland N, Tillung RH, et al. Protective measures against depredation on sheep: shepherding and use of livestock guardian dogs in Lierne. Final report - 2000. 2000.

61. Lance NJ. Application of electrified fladry to desreask risk of livestock depredation by wolves (*Canis lupus*). Logan: Utah State University; 2009.

62. Landa A, Gudvangen K, Swenson JE, Røskaft E. Factors associated with wolverine *Gulo gulo* predation on domestic sheep. Journal of Applied Ecology. 1999;36(6):963-73. doi: 10.1046/j.1365-2664.1999.00451.x.

63. Landry J-M, Raydelet P. Efficacité des chiens de protection contre la prédation du lynx dans le Massif jurassien: Présentation préliminaire des résultats de l’enquête de terrain. Lons le Saunier: Pôle Grands Prédateurs, 2010 Juin 2010. Report No.

64. Leigh J. Effects of aversive conditioning on behavior of nuisance Louisiana black bears. Baton Rouge: Louisiana State University and Agricultural and Mechanical College; 2007.

65. Lichtenfeld LL, Trout C, Kisimir EL. Evidence-based conservation: predator-proof bomas protect livestock and lions. Biodiversity and Conservation. 2015;24(3):483-91. doi: 10.1007/s10531-014-0828-x.

66. Linhart SB, Roberts JD, Dasch GJ. Electric fencing reduces coyote predation on pastured sheep. Journal of Range Management. 1982;35:276-81.

67. Linhart SB, Sterner RT, Dasch GJ, Theade JW. Efficacy of light and sound stimuli for reducing coyote predation upon pastured sheep. Protection Ecology. 1984;6:75-84.

68. Linhart SB, Dasch GJ, Johnson RB, Roberts JD, Packham CJ, editors. Electronic frightening devices for reducing coyote predation on domestic sheep: efficacy under range conditions and operational use. Proceedings of the Fifteenth Vertebrate Pest Conference; 1992; Davis: University of California.

69. Maclennan SD, Groom RJ, Macdonald DW, Frank LG. Evaluation of a compensation scheme to bring about pastoralist tolerance of lions. Biological Conservation. 2009;142(11):2419-27. doi:10.1016/j.biocon.2008.12.003.

70. Mahoney S, Charry AA. The use of alpacas as new-born lamb protectors to minimise fox predation. Extension Farming Systems Journal. 2007;1(1):65-70.

71. Marker LL, Dickman AJ, Macdonald DW. Survivorship and causes of mortality for livestock-guarding dogs on Namibian rangeland. Rangeland Ecology & Management. 2005;58(4):337-43. doi: 10.2111/1551-5028(2005)058[0337:SACOMF]2.0.CO;2.

72. Martin J, O'Brien A. The use of bone oil (Renadine) as a coyote repellent on sheep farms in Ontario. Proceedings of the 19th Vertebrate Pest Conference. 2000:310-1.

73. Mazzolli M, Graipel ME, Dunstone N. Mountain lion depredation in southern Brazil. Biological Conservation. 2002;105(1):43-51. doi: 10.1016/S0006-3207(01)00178-1.

74. McManus JS, Dickman AJ, Gaynor D, Smuts BH, Macdonald DW. Dead or alive? Comparing costs and benefits of lethal and non-lethal human-wildlife conflict mitigation on livestock farms. Oryx. 2015;49(4):687-95. doi: 10.1017/S0030605313001610.

75. Meadows LE, Knowlton FF. Efficacy of guard llamas to reduce canine predation on domestic sheep. Wildlife Society Bulletin (1973-2006). 2000;28(3):614-22.

76. Mech LD, Harper EK, Meier TJ, Paul WJ. Assessing factors that may predispose Minnesota farms to wolf depredations on cattle. Wildlife Society Bulletin. 2000;28(3):623-9.

77. Michalski F, Boulhosa RLP, Faria A, Peres CA. Human–wildlife conflicts in a fragmented Amazonian forest landscape: determinants of large felid depredation on livestock. Animal Conservation. 2006;9(2):179-88. doi: 10.1111/j.1469-1795.2006.00025.x.

78. Miller GD. Field tests of potential polar bear repellents. Bears: Their Biology and Management, A Selection of Papers from the Seventh International Conference on Bear Research and Management; Williamsburg VA, USA, and Plitvice Lakes, Yugoslavia: International Assocation for Bear Research and Management; 1987. p. 383-90.

79. Mitchell BR, Jaeger MM, Barrett RH. Coyote depredation management: current methods and research needs. Wildlife Society Bulletin. 2004;32(4):1209-18. doi: 10.2193/0091-7648(2004)032[1209:CDMCMA]2.0.CO;2

80. Musiani M, Mamo C, Boitani L, Callaghan C, Gates CC. Wolf depredation trends and the use of fladry barriers to protect livestock in western North America. Conservation Biology. 2003;17(6):1238-547. doi: 10.1111/j.1523-1739.2003.00063.x.

81. Nass RD, Theade J. Electric fences for reducing sheep losses to predators. Journal of Range Management. 1988;41:251-2.

82. National Project Steering Committee. National wild dog action plan: promoting and supporting community-driven action for landscape-scale wild dog management. Barton, ACT: WoolProducers Australia, 2014.

83. Obbard ME, Howe EJ, Wall LL, Allison B, Black R, Davis P, et al. Relationships among food availability, harvest, and human–bear conflict at landscape scales in Ontario, Canada. Ursus. 2014;25(2):98-110. doi: 10.2192/URSUS-D-13-00018.1.

84. Odden J, Herfindal I, Linnell JDC, Andersen R. Vulnerability of domestic sheep to lynx depredation in relation to roe deer density. Journal of Wildlife Management. 2008;72(1):276-82. doi: 10.2193/2005-537.

85. Odden J, Nilsen EB, Linnell JDC. Density of Wild Prey Modulates Lynx Kill Rates on Free-Ranging Domestic Sheep. PLOS ONE. 2013;8(11):e79261. doi: 10.1371/journal.pone.0079261.

86. Ogada MO, Woodroffe R, Oguge NO, Frank LG. Limiting depredation by African carnivores: the role of livestock husbandry. Conservation Biology. 2003;17(6):1521-30. doi: 10.1111/j.1523-1739.2003.00061.x.

87. Otstavel T, Vuori K, A., Sims DE, Valros A, Vainio O, Saloniemi H. The first experience of livestock guarding dogs preventing large carnivore damages in Finland. Estonian Journal of Ecology. 2009;58(3):216. doi: 10.3176/eco.2009.3.06.

88. Palmer BC, Conover MR, Frey SN. Replication of a 1970s study on domestic sheep losses to predators on Utah's summer rangelands. Rangeland Ecology & Management. 2010;63(6):689-95. doi: 10.2111/REM-D-09-00190.1.

89. Peebles KA, Wielgus RB, Maletzke BT, Swanson ME. Effects of Remedial Sport Hunting on Cougar Complaints and Livestock Depredations. PLoS ONE. 2013;8(11):e79713. doi: 10.1371/journal.pone.0079713.

90. Rigg R, Finďo S, Wechselberger M, Gorman ML, Sillero-Zubiri C, Macdonald DW. Mitigating carnivore–livestock conflict in Europe: lessons from Slovakia. Oryx. 2011;45(02):272-80. doi: 10.1017/S0030605310000074.

91. Rossler ST, Gehring TM, Schultz RN, Rossler MT, Wydeven AP, Hawley JE. Shock collars as a site-aversive conditioning tool for wolves. Wildlife Society Bulletin. 2012;36(1):176-84. doi: 10.1002/wsb.93.

92. Rust NA, Whitehouse-Tedd KM, MacMillan DC. Perceived efficacy of livestock-guarding dogs in South Africa: implications for cheetah conservation. Wildlife Society Bulletin. 2013;37(4):690-7. doi:10.1002/wsb.352.

93. Sagør JT, Swenson JE, Røskaft E. Compatibility of brown bear *Ursus arctos* and free-ranging sheep in Norway. Biological Conservation. 1997;81(1):91-5. doi: 10.1016/S0006-3207(96)00165-6.

94. Salvatori V, Mertens AD. Damage prevention methods in Europe: experiences from LIFE nature projects. Hystrix, the Italian Journal of Mammalogy. 2012;23(1):73-9. doi: 10.4404/hystrix-23.1-4548.

95. Sampson FW, Brohn A. Missouri's program of extension predator control. The Journal of Wildlife Management. 1955;19(2):272-80. doi: 10.2307/3796863.

96. Schultz RN, Jonas KW, Skuldt LH, Wydeven AP. Experimental use of dog-training shock collars to deter depredation by gray wolves. Wildlife Society Bulletin. 2005;33(1):142-8. doi: 10.2193/0091-7648(2005)33[142:EUODSC]2.0.CO;2.

97. Shivik JA, Treves A, Callahan P. Nonlethal techniques for managing predation: primary and secondary repellents. Conservation Biology. 2003;17(6):1531-7. doi: 10.1111/j.1523-1739.2003.00062.x.

98. Stahl P, Vandel JM, Herrenschmidt V, Migot P. The effect of removing lynx in reducing attacks on sheep in the French Jura Mountains. Biological Conservation. 2001;101(1):15-22. doi: 10.1016/S0006-3207(01)00054-4.

99. Stahl P, Vandel JM, Ruette S, Coat L, Coat Y, Balestra L. Factors affecting lynx predation on sheep in the French Jura. Journal of Applied Ecology. 2002;39(2):204-16. doi: 10.1046/j.1365-2664.2002.00709.x.

100. Stander PE. A suggested management strategy for stock-raiding lions in Namibia. South African Journal of Wildlife Research - 24-month delayed open access. 1990;20(2):37-43.

101. Suryawanshi KR, Bhatnagar YV, Redpath S, Mishra C. People, predators and perceptions: patterns of livestock depredation by snow leopards and wolves. Journal of Applied Ecology. 2013;50(3):550-60. doi: 10.1111/1365-2664.12061.

102. Swanson VB, Scott GE. Livestock protectors for sheep predator control. Proceedings, Western Section, American Society of Animal Science. 1973;24:34-6.

103. Treves A, Martin KA, Wydeven AP, Wiedenhoeft JE. Forecasting environmental hazards and the application of risk maps to predator attacks on livestock. BioScience. 2011;61(6):451-8. doi: 10.1525/bio.2011.61.6.7.

104. Tumenta PN, de Iongh HH, Funston PJ, Udo de Haes HA. Livestock depredation and mitigation methods practised by resident and nomadic pastoralists around Waza National Park, Cameroon. Oryx. 2013;47(2):237-42. Epub 2013/04/01. doi: 10.1017/S0030605311001621.

105. Valeix M, Hemson G, Loveridge AJ, Mills G, Macdonald DW. Behavioural adjustments of a large carnivore to access secondary prey in a human-dominated landscape. Journal of Applied Ecology. 2012;49:73-81. doi: 10.1111/j.1365-2664.2011.02099.x.

106. van Bommel L. Guardian dogs for livestock protection in Australia. Hobart: University of Tasmania; 2013.

107. van Bommel L, Johnson CN. Good dog! Using livestock guardian dogs to protect livestock from predators in Australia’s extensive grazing systems. Wildlife Research. 2012;39(3):220-9. doi: 10.1071/WR11135.

108. van Bommel L, Bij de Vaate MD, De Boer WF, De Iongh HH. Factors affecting livestock predation by lions in Cameroon. African Journal of Ecology. 2007;45(4):490-8. doi: 10.1111/j.1365-2028.2007.00759.x.

109. van Liere D, Dwyer C, Jordan D, Premik-Banič A, Valenčič A, Kompan D, et al. Farm characteristics in Slovene wolf habitat related to attacks on sheep. Applied Animal Behaviour Science. 2013;144(1):46-56. doi: 10.1016/j.applanim.2012.12.005.

110. Wagner KK, Conover MR. Effect of preventive coyote hunting on sheep losses to coyote predation. The Journal of Wildlife Management. 1999;63(2):606-12. doi: 10.2307/3802649.

111. Walking for Lions. First quarterly report. Pandamatenga, Botswana: Walking for Lions: wild lion protection & survival, 2016.

112. Wilson SM, Madel MJ, Mattson DJ, Graham JM, Burchfield JA, Belsky JM. Natural landscape features, human-related attractants, and conflict hotspots: a spatial analysis of human–grizzly bear conflicts. Ursus. 2005;16(1):117-29. doi: 10.2192/1537-6176(2005)016[0117:NLFHAA]2.0.CO;2.

113. Woodroffe R, Frank LG, Lindsey PA, ole Ranah SMK, Romañach S. Livestock husbandry as a tool for carnivore conservation in Africa's community rangelands: a case-control study. Biodiversity and Conservation. 2007;16(4):1245-60. doi: 10.1007/s10531-006-9124-8.

114. Woodroffe R, Lindsey P, Romañach S, Stein A, ole Ranah SMK. Livestock predation by endangered African wild dogs (*Lycaon pictus*) in northern Kenya. Biological Conservation. 2005;124(2):225-34. doi: 10.1016/j.biocon.2005.01.028.

115. Wooldridge DR. Polar bear electronic derrent and detection systems. Bears: Their Biology and Management, A Selection of Papers from the Fifth International Conference on Bear Research and Management; February 1980; Madison WI, USA: International Assocation for Bear Research and Management; 1983. p. 264-9.

116. Zarco-González MM, Monroy-Vilchis O. Effectiveness of low-cost deterrents in decreasing livestock predation by felids: a case in Central Mexico. Animal Conservation. 2014;17(4):371-8. doi: 10.1111/acv.12104.
