# Supplementary figures and images for: Carnivore conservation needs evidence-based livestock protection
Source: PLoS Biol. 2018 Sep 18;16(9):e2005577. doi: 10.1371/journal.pbio.2005577 (PMC6143182; doi:10.1371/journal.pbio.2005577)

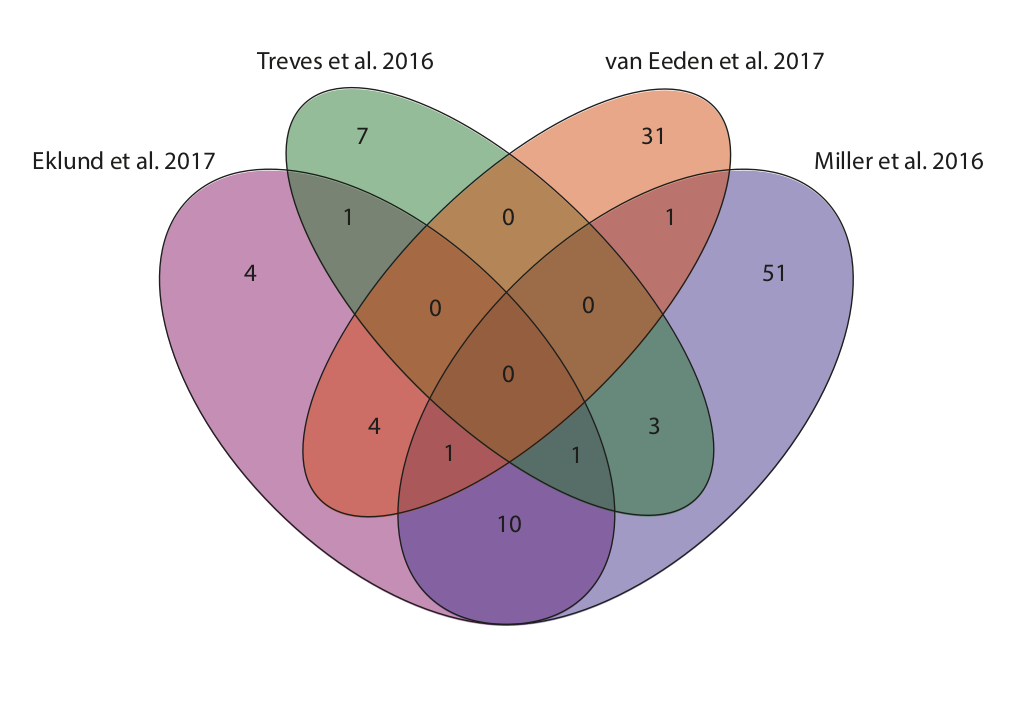

Supplement: S1 Fig — (TIF) [file pbio.2005577.s003.tif]
